# A Digital Registry and Education Network for Child Maltreatment Protection: Protocol for a Pilot Observational Exploratory Intervention Study

**DOI:** 10.2196/89128

**Published:** 2026-09-02

**Authors:** Gianvincenzo Zuccotti, Dario Dilillo, Antonella Agosto, Valeria Brazzoduro, Eloisa Brunilde Lina Marinelli, Valeria Calcaterra

**Affiliations:** 1 Department of Biomedical and Clinical Sciences University of Milan Milano, Lombardy Italy; 2 Department of Pediatrics Ospedale dei Bambini Vittore Buzzi Milano, Lombardy Italy; 3 Azienda Socio-Sanitaria Territoriale Fatebenefratelli Sacco Milano, Lombardy Italy; 4 Department of Internal Medicine and Therapeutics University of Pavia Pavia, Lombardy Italy

**Keywords:** digital registry, education network, child maltreatment, protection, children

## Abstract

**Background:**

Child maltreatment, a major public health concern, has long-term neurobiological, psychological, and social consequences. Recognizing, documenting, and reporting suspected cases should be systematic across hospital and community services, but these processes are hindered by inconsistent surveillance systems, uneven professional training, and the lack of standardized data collection tools. Timely follow-up, updated clinical criteria, and structured multidisciplinary training can improve case assessment, traceability, and professionals’ ability to report suspected maltreatment. The Sentinel project was developed to address these clinical, organizational, and public health gaps through a combined REDCap-based digital registry and structured training program for pediatric health care professionals.

**Objective:**

This study aims to evaluate the usability, feasibility, and preliminary impact of an integrated surveillance and training system designed to improve the early detection, documentation, and reporting of suspected child maltreatment by pediatricians and health care professionals.

**Methods:**

This is an observational, exploratory, monocentric pilot implementation study with a pretest-posttest evaluation design. The study will be conducted over 24 months in a hospital-community pediatric network. Voluntary participants will be hospital and community pediatricians and other pediatric health care professionals who provide written informed consent. The project includes two interconnected components: (1) implementation of a secure, anonymized REDCap registry for standardized documentation of suspected maltreatment, follow-up, reporting pathways, and case management outcomes and (2) a theoretical-practical training program delivered through lectures, e-learning modules, webinars, supervised exercises, and hands-on registry sessions. Primary and secondary outcomes will be assessed at prespecified time points, including baseline, immediately after training, during registry use, and at the end of follow-up. Registry usability will be measured using the System Usability Scale; training effectiveness will be measured through pretest-posttest knowledge, competency tests, and satisfaction questionnaires; and reporting outcomes will be measured through comparison of pre- and postimplementation reporting rates.

**Results:**

The study will report the usability, feasibility, and preliminary implementation outcomes of the integrated registry training system. Main results will include System Usability Scale scores, registry completion indicators, reporting timeliness, the number and rate of suspected maltreatment reports before and after implementation, changes in knowledge and competency scores, participant satisfaction, and qualitative feedback on barriers to and facilitators of implementation. Participant recruitment and data collection began in April 2026 and are currently ongoing. Completion of data collection and publication of the results are expected between December 2027 and January 2028.

**Conclusions:**

The Sentinel project will test an innovative and scalable pilot model that integrates digital surveillance with structured professional training to enhance the early detection, documentation, and management of suspected child maltreatment. By standardizing data collection, strengthening professional competencies, and fostering collaboration across hospital and community settings, the study will inform the development of a regional or national observatory and support an evidence-based, system-wide approach to child protection.

**Trial Registration:**

ClinicalTrials.gov NCT07250074; https://clinicaltrials.gov/study/NCT07250074

**International Registered Report Identifier (IRRID):**

PRR1-10.2196/89128

## Introduction

### Theoretical Context and Framing of the Problem

Child maltreatment and abuse represent one of the most serious and persistent public health and social emergencies of the 21st century, with profound repercussions on neurobiological development, emotional regulation, mental health, physical health, social functioning, and life-course well-being [1-14]. These consequences make early identification and timely protection a core responsibility of pediatric and public health systems rather than a problem confined to social or judicial services.

The World Health Organization defines child maltreatment as any form of physical, sexual, or psychological abuse, neglect, or exploitation that results in actual or potential harm to the child’s health, survival, or dignity [1,12]. According to the *Global status report on preventing violence against children 2020* [13], approximately 1 billion children worldwide experience some form of violence each year. The consequences of such experiences often persist into adulthood, increasing the risk of psychiatric disorders, chronic diseases, and relational dysfunctions [2-6].

Available national and institutional data show that child maltreatment remains difficult to quantify, with recorded cases representing only the visible part of a largely underestimated phenomenon [15-18]. Underrecognition, delayed reporting, heterogeneous procedures, and limited surveillance systems hinder health services’ ability to monitor suspected cases, describe their epidemiology, and evaluate the timeliness and effectiveness of protective responses [15-18].

Scientific literature demonstrates that early exposure to violence or neglect induces lasting alterations in neuroendocrine circuits and in the regulation of stress responses [19-24]. Witnessed violence, in which a child is exposed to aggressive acts within the family environment, produces psychological effects comparable to those observed in direct survivors [25-27]. These findings emphasize the urgent need for early, integrated, and data-driven interventions aimed at prevention, identification, and timely protection.

### State of the Art and Operational Gaps

Despite significant legislative advances and an increasing cultural awareness of child protection, the management of child maltreatment in Italy remains heterogeneous and fragmented [17]. Reporting procedures vary widely across regions and services, and the absence of a unified information system hampers both the epidemiological analysis of cases and the assessment of intervention effectiveness.

Health care professionals, particularly pediatricians, occupy a pivotal position in the early identification of abuse and neglect because they may observe sentinel injuries, developmental concerns, psychosomatic symptoms, family risk factors, or repeated access to care before other agencies become involved [28-31]. Nevertheless, recognition and reporting require not only clinical awareness but also shared definitions, standardized documentation, clear legal-operational pathways, and confidence in interprofessional communication. The absence of these supports contributes to underreporting; delayed notification; incomplete documentation; and inconsistent coordination among hospitals, community pediatrics, social services, and child protection authorities [17,19,28-32].

At the international level, clinical registries have been used to improve data quality, case traceability, auditability, and the monitoring of care pathways, particularly when data items are standardized and embedded in routine clinical workflows [33-36]. In parallel, structured and multidisciplinary educational programs have been associated with improvements in knowledge, recognition skills, confidence, and reporting behavior among health care professionals involved in child protection [37-43]. These findings support an integrated model in which digital infrastructure and professional education are implemented together rather than as isolated quality improvement activities.

Nevertheless, in Italy, there is still no established model that systematically combines technological innovation, professional training, and territorial governance in addressing child maltreatment. The Sentinel project seeks to fill this critical gap by integrating these dimensions into a unified and evidence-based framework capable of supporting both clinical practice and policy development.

### Hypothesis and Rationale of the Project

In light of these critical issues, the Sentinel project was conceived to create an integrated surveillance and training system for the early recognition of suspected child maltreatment, combining technological innovation, professional education, and hospital-community coordination.

The underlying hypothesis is that the combination of a standardized digital registry and a structured training program for pediatricians and health care professionals can significantly improve the capacity of the health care system to identify, document, and respond to cases of suspected maltreatment. Specifically, the project assumes that this integrated approach will (1) enhance the early detection of suspected child abuse and neglect; (2) improve the quality, completeness, and timeliness of reporting; and (3) strengthen collaboration between hospital and community-based services, thereby increasing the efficiency of multidisciplinary care for vulnerable children.

The registry, developed using the REDCap platform (Vanderbilt University), will enable the anonymous and standardized collection of clinical and social data while ensuring data security, traceability, and compliance with privacy regulations.

In parallel, the training component, structured through theoretical and practical modules, will provide health care professionals with the clinical, communication, legal, and operational skills required to recognize warning signs, activate reporting pathways, document case information, and use the registry appropriately. This dual approach is designed to promote a shared language and a consistent, evidence-based methodology among pediatric and allied health professionals.

### Scientific Significance and Expected Impact

The Sentinel project adopts a translational research perspective, aiming to transform scientific evidence into operational tools and effective prevention policies. By integrating data collection with continuous professional training, the project bridges the gap between research and clinical practice, promoting the direct application of evidence to improve child protection.

Sentinel aspires to validate a replicable model of clinical-epidemiological surveillance of child maltreatment capable of generating robust data for health planning and fostering a cultural shift within community pediatrics toward proactive identification and intervention. The integration of technology and education establishes an innovative paradigm in which digital systems support professional empowerment and, ultimately, the protection of children at risk.

In the long term, this initiative aims to lay the groundwork for the establishment of a regional observatory on child maltreatment with the potential for national expansion. Such an observatory would provide a sustainable infrastructure for continuous surveillance, professional education, and policy development, ensuring that child protection becomes a structural and data-informed component of public health systems.

### Aim of the Study

The aim of this study is to evaluate the usability, feasibility, and preliminary implementation impact of the Sentinel digital registry and training network in improving standardized case identification, documentation quality, reporting timeliness, and case management among hospital and community pediatric health care professionals.

## Methods

### Study Design

The Sentinel study is an observational, exploratory, monocentric pilot implementation study with a pretest-posttest evaluation design and an overall duration of 24 months. The project does not introduce an experimental clinical treatment and does not modify the legal or clinical duties of professionals involved in suspected child maltreatment. Rather, it evaluates the implementation of 2 organizational tools: a secure digital registry for standardized documentation and a structured training program for pediatric health care professionals. The primary objective is to assess registry usability and feasibility. Secondary objectives are to evaluate training effectiveness, reporting completeness, reporting timeliness, and preliminary changes in suspected maltreatment reporting rates before and after implementation.

The project is organized into 2 interconnected components: the registry component and the training component. These components will be implemented sequentially and then evaluated jointly because the registry is intended to standardize documentation, whereas training is intended to improve recognition, case management confidence, and appropriate use of the registry.

The operational sequence will include development and internal testing of the REDCap registry; definition of the minimum dataset and reporting workflow; recruitment of hospital and community pediatric professionals; baseline assessment of knowledge, competencies, and reporting activity; delivery of the training program; activation of registry data entry; continuous monitoring of registry completeness and technical issues; follow-up assessment of outcomes; statistical analysis; and dissemination of results. All activities will be coordinated by an internal scientific committee responsible for methodological quality, ethical compliance, data protection procedures, and adherence to project timelines.

Because of its observational and pilot implementation nature, the study will not interfere with clinical decision-making, child protection duties, or mandatory reporting obligations. Professionals will continue to follow existing institutional and legal procedures for suspected maltreatment. Study data will be used only to evaluate registry use, reporting activity, and educational outcomes and identify preliminary indicators of feasibility, usability, acceptability, and implementation impact that can inform future multicenter or regional surveillance models.

### Study Setting and Timeline

The study will be conducted within the coordinating pediatric hospital and its connected community pediatric network. The 24-month timeline will include approximately 3 months for registry development and internal testing, 3 months for recruitment and baseline assessment, 3 months for training delivery, 12 months for registry activation and follow-up data collection, and 3 months for final analysis and dissemination. Minor adaptations to these periods may be made according to ethics approval timelines and operational feasibility.

### Participants and Recruitment

The study population will consist of health care professionals from the regional pediatric network, including hospital and community pediatricians, pediatric residents, nurses, psychologists, and other professionals involved in the recognition, documentation, or management of suspected child maltreatment according to local organizational arrangements.

Recruitment will occur through institutional invitations circulated within the hospital-community pediatric network. Participation will be voluntary and require written informed consent. Eligible participants will be professionals aged 18 years or older who work in pediatric or child health settings, attend the training program, and agree to complete the evaluation questionnaires. Participants will be asked to complete baseline and posttraining assessments and, where applicable, use the registry during the implementation period.

Exclusion criteria will comprise refusal or withdrawal of consent, failure to complete the training program, nonparticipation in the practical registry sessions, or missing primary outcome data for the relevant analysis. A minimum of 30 professionals will be targeted. This sample is considered appropriate for a pilot feasibility study and for preliminary estimation of usability, training gains, and reporting process indicators.

### Implementation Components and Procedures

#### Registry Component

The digital registry will be developed using the REDCap platform, a system widely used in clinical research for secure data collection, management, and analysis. The registry will be configured with role-based access, audit trails, structured data entry fields, mandatory and optional variables, and data quality checks. Access will be restricted to authorized project personnel, and all data management procedures will comply with applicable institutional policies and data protection regulations.

The registry will collect only anonymized or non–directly identifiable information required for research and service evaluation. Data fields will include child age group and sex, date and setting of contact, type of suspected maltreatment (physical, psychological, or sexual maltreatment; neglect; or exposure to domestic violence), warning signs, risk and protective factors, reporting source, referral or follow-up arrangements, and case management outcome. Each record will be assigned a unique alphanumeric study code. Direct identifiers, such as name, fiscal code, address, telephone number, or full date of birth, will not be entered into the research registry. Potential duplicate reports will be managed through predefined nonidentifying linkage variables, including time window, age group, sex, setting, and event characteristics, without attempting to reidentify the child.

Registry usability will be evaluated using the System Usability Scale (SUS), a validated 10-item questionnaire yielding scores from 0 to 100 [44]. SUS completion will be restricted to trained and authorized professionals with documented exposure to the registry, as detailed in the Outcomes and Measurement Time Points section. Feasibility indicators will include number of active users, number of records created, proportion of completed mandatory fields, average time required to complete a report, number of technical support requests, proportion of records with documented pathway activation, and completeness of follow-up fields. These indicators will allow for evaluation of whether the registry reduces data fragmentation and supports standardized clinical and operational terminology.

The impact of the integrated registry training system on suspected maltreatment reporting will be assessed by comparing pre- and postimplementation reporting rates. The numerator will be the number of suspected maltreatment reports recorded during each observation period. The denominator will be the number of participating professionals and, when available, the number of pediatric consultations or service contacts during the same period. Reporting rates will therefore be expressed as reports per professional month and, where denominators are available, reports per 1000 pediatric contacts.

#### Training Component

Training is a strategic component of the project and is designed as an integrated educational intervention with theoretical, practical, and supervised components. Its purpose is to improve recognition of suspected maltreatment, appropriate documentation, awareness of legal and ethical responsibilities, confidence in multidisciplinary communication, and correct use of the REDCap registry.

The theoretical phase will include in-person lectures and e-learning modules. Training content will cover definitions and typologies of maltreatment, clinical warning signs, differential diagnoses, trauma-informed communication with children and families, documentation standards, legal and institutional reporting pathways, interprofessional coordination, data protection, and correct use of the digital registry.

The practical phase will include online and interactive webinars; hands-on sessions on data entry into the registry; and small-group supervision sessions led by experts in pediatrics, psychology, and forensic medicine. The effectiveness of the training will be evaluated using a pretest-posttest repeated-measure design. Each participant will complete a knowledge and competency assessment questionnaire before and after the training. The questionnaire will include multiple-choice questions and clinical scenarios aimed at measuring participants’ ability to recognize indicators of abuse, activate appropriate reporting pathways, and use the digital tool effectively.

Participants’ satisfaction, perceived usefulness, confidence, and perceived transferability to clinical practice will be assessed after training using 5-point Likert-scale items and open-ended questions. Results will be summarized using descriptive statistics and complemented with thematic qualitative analysis of open-ended responses to identify perceived strengths, implementation barriers, training needs, and suggestions for improvement.

Exploratory multivariable analyses will identify predictors of training gain, satisfaction, registry usability, and active registry use considering age, professional role, years of experience, work setting, previous child protection training, and frequency of registry use.

### Outcomes and Measurement Time Points

The primary outcome is registry usability, measured using the mean SUS score collected after participants use the registry. Specifically, the SUS will be administered once at the end of the 12-month registry activation and follow-up period or earlier for participants who discontinue participation after having completed the minimum required registry exposure. Eligible respondents will be trained and authorized hospital or community professionals involved in suspected maltreatment recognition, documentation, or case management. To ensure that SUS responses reflect actual exposure to the tool, participants will be included in the primary SUS analysis only if they have entered at least one registry record or completed at least one supervised registry use session or simulation. Participants without any registry exposure will not contribute to the primary SUS analysis.

Secondary outcomes include feasibility indicators (active users, number of records, completion of mandatory fields, technical support requests, and time to complete a report), training outcomes (pretest-posttest knowledge and competency scores, satisfaction, and qualitative feedback), and reporting outcomes (number and rate of suspected maltreatment reports, reporting timeliness, duplicate reports, follow-up documentation, and activation of child protection pathways). Baseline data will be collected before training and registry activation, posttraining outcomes will be collected immediately after training, and registry usability and implementation outcomes will be collected during registry use and at the end of the observation period.

### Data Management, Confidentiality, and Registry Workflow

Registry data will be entered using predefined forms by trained and authorized professionals. Records will be entered by the professional who identifies or manages the suspected case or by another authorized member of the clinical team directly involved in the case according to local organizational arrangements. Follow-up information will be updated when new nonidentifying information becomes available regarding referral, pathway activation, multidisciplinary evaluation, or case management outcome. Records will be saved under a unique study code and stored on secure institutional servers or approved REDCap infrastructure. The registry will not contain direct identifiers. Data access will be role based and limited to authorized members of the research team. Data exports for analysis will be anonymized, stored in password-protected files, and used only for the objectives approved by the ethics committee. Data quality will be checked periodically by designated members of the research team through REDCap validation rules, completeness checks, audit trails, and review of mandatory fields. Any inconsistencies, missing mandatory information, or potential duplicate records will be resolved using only nonidentifying registry information. Potential duplicate reports will be assessed through predefined nonidentifying linkage variables, including time window, age group, sex, setting, and event characteristics. When a potential duplicate is detected, the research team will retain a single registry record for analysis and update it with additional nonidentifying information when appropriate. Duplicate management will not involve access to direct identifiers and will not interfere with clinical or legal reporting procedures.

Any suspected maltreatment identified in clinical practice will continue to be managed through ordinary institutional and legal reporting pathways; the registry is an evaluation and documentation tool and does not replace mandatory reporting procedures. Mandatory reporting will remain a separate clinical and legal process carried out through existing institutional channels and according to applicable law. Entry of information into the research registry will be separate from the activation of the appropriate clinical and legal pathway and will not be considered a substitute for mandatory reporting. No direct identifiers or legal reporting documents will be uploaded to the registry.

### Statistical Analysis

#### Overview

Quantitative analyses will be performed using recognized statistical software, such as SPSS, R, or Stata. Descriptive statistics will be calculated for all study variables. Continuous variables will be summarized as means and SDs or medians and IQRs according to distribution. Categorical variables will be summarized as frequencies and percentages. All estimates will be reported with 95% CIs when appropriate.

Pretest-posttest comparisons of knowledge and competency scores will be performed using 2-tailed paired-sample *t* tests when normality assumptions are met, as assessed using the Shapiro-Wilk test and visual inspection of distributions. When assumptions are not met, Wilcoxon signed-rank tests will be used. Mean change scores, standardized effect sizes, and 95% CIs will be reported to support interpretation of training effectiveness.

Registry usability will be evaluated by calculating the mean SUS score and comparing it with established interpretive benchmarks, where scores of 70 or higher are considered acceptable and scores of 80 or higher indicate excellent usability [44]. A 1-sample *t* test or Wilcoxon signed-rank test will be used to compare observed SUS scores with the prespecified benchmark depending on distributional characteristics.

Changes in reporting rates of suspected child maltreatment before and after implementation will be analyzed using Poisson regression models, estimating incidence rate ratios with 95% CIs. The primary pretest-posttest comparison will use reports per professional month as the operational denominator. When service volume data are available, additional models will use the number of pediatric consultations or contacts as an offset to estimate reports per 1000 contacts. Overdispersion will be assessed by comparing the deviance and Pearson chi-square statistics with the df; if overdispersion is present, negative binomial regression or robust SEs will be applied. Repeated reports and potential duplicates will be described separately and, in sensitivity analyses, excluded or counted once per suspected case according to the predefined duplicate management rules.

Associations between participant characteristics and outcomes will be explored using multivariable linear, logistic, or count regression models according to the distribution and nature of the dependent variable. Candidate predictors will include professional role, years of experience, work setting, previous child protection training, participation in practical sessions, and frequency of registry use. All analyses will be 2 tailed, with a significance level set at *P*<.05.

Missing data will be described by variable and time point. Complete-case analysis will be used for the primary pilot analyses. If the proportion and pattern of missing data justify it, multiple imputation or sensitivity analyses will be performed. Qualitative responses will be analyzed thematically by grouping open-text answers into recurrent categories related to usability, workflow integration, training usefulness, barriers, and facilitators.

#### Sample Size Calculation

The sample size was determined to ensure adequate statistical power for detecting meaningful effects in both the usability and training evaluations. Assuming a medium effect size (Cohen *d*=0.5) for the improvement in knowledge and competency scores between pre- and posttraining assessments, a paired-sample *t* test with an α level of .05 and power (1 – β) of 0.80 requires a minimum of 27 participants.

This estimate aligns with the planned recruitment target of 25 to 30 professionals, which is considered sufficient to assess feasibility, usability, and preliminary impact within this exploratory pilot phase. For the SUS usability analysis, a sample of 25 participants allows for the estimation of the mean score with a 95% CI width of ±5 points, which is consistent with published methodological recommendations for usability testing in health care settings. Because this is a monocentric pilot study, analyses will be interpreted primarily as estimates of feasibility and effect size for future multicenter studies rather than as definitive evidence of effectiveness.

### Ethical Considerations

The Sentinel project will be conducted in full accordance with the Declaration of Helsinki, relevant national regulations, institutional policies, and the European General Data Protection Regulation (Regulation [EU] 2016/679).

All participating health care professionals will receive detailed written information describing the study aims and procedures, voluntary nature of participation, data collected, confidentiality safeguards, and right to withdraw without consequences. Written informed consent will be obtained before participation in training evaluations, usability assessments, and research data analysis.

The registry will not collect direct identifiers of children, families, or caregivers. Only anonymized or non–directly identifiable variables necessary for research and service evaluation will be entered. The registry will be separated from clinical records and will not replace institutional case files or mandatory legal reporting. Information collected for the study will be used exclusively for research, audit, and service improvement purposes approved by the ethics committee without any adverse clinical or administrative consequences for children or families.

The project protocol will be submitted for review and approval by the ethics committee of the coordinating hospital (Comitato Etico Territoriale [CET] Lombardia Area 1_2025). Participation in the study does not entail direct clinical risk for professionals and does not add costs for the health care system beyond planned project activities. Because the topic involves highly sensitive child protection information, access privileges, audit trails, password protection, secure storage, and anonymized exports will be used to minimize privacy and confidentiality risks.

All training and data collection activities will be conducted in a manner that protects dignity, safety, privacy, and confidentiality. Mandatory reporting obligations and urgent child protection actions will remain governed by existing laws and institutional procedures; participation in the Sentinel project will not delay or modify any duty to report suspected maltreatment.

The study protocol was registered in ClinicalTrials.gov (ID NCT07250074). If modifications to the original protocol are required, amendments will be submitted to the ethics committee for approval before implementation, and the approved information will be updated on ClinicalTrials.gov.

To ensure greater scientific rigor, the manuscript was prepared in accordance with the SPIRIT (Standard Protocol Items: Recommendations for Interventional Trials) guidelines for reporting study protocols.

## Results

The study was funded in September 2025, and ethical approval was obtained in January 2026. Participant recruitment and data collection began in April 2026 and are currently ongoing. Data analysis has not yet started. Completion of data collection and publication of the results are expected between December 2027 and January 2028.

### Registry Phase Results

The registry component will generate implementation results on the use of the REDCap platform for systematic, secure, and standardized recording of suspected child maltreatment reports [45]. Reported results will include the number of trained users, number of active registry users, number of records created, completion of mandatory fields, time required for report completion, and number of technical or workflow issues identified during implementation.

The analysis will describe whether the registry provides a more unified view of suspected cases and care pathways than the preimplementation documentation process. Completeness, traceability, documented actions, follow-up fields, and pathway activation will be used as operational indicators of report quality.

Registry usability will be described using SUS scores, reported as means, SDs, medians, IQRs, 95% CIs, and the proportion of scores below 70 and above 80 [44]. To evaluate implementation, reporting rates will be compared before and after registry introduction and expressed as reports per professional month and, where denominators are available, per 1000 pediatric contacts.

The registry will describe suspected cases by maltreatment type, child characteristics, family and social context, access route, setting, referral pathway, follow-up, and outcome. These data will help assess whether the model can generate useful local information to support prevention strategies, service indicators, and health care planning.

### Training Phase Results

The analysis will examine whether theoretical and practical training improves participants’ ability to recognize early warning signs of abuse or neglect, communicate with children and families using trauma-informed principles, select appropriate reporting procedures, and complete the registry accurately.

The training evaluation will report Likert-scale satisfaction scores, perceived usefulness, perceived transferability to clinical practice, and confidence in using the registry [46]. Open-ended responses will be analyzed thematically to identify perceived strengths; barriers; suggestions for improvement; and key issues related to clinical relevance, content clarity, registry usability, reporting procedures, and coordination with social and child protection services. These qualitative data will complement quantitative outcomes and support refinement of the training model.

Overall, the training results will indicate whether the project can consolidate a network of informed and coordinated professionals, so-called Sentinel pediatricians, capable of supporting surveillance activities and sustaining standardized procedures beyond the initial pilot phase.

### Overall Evaluation Outcomes

Integrated statistical analysis of registry and training data will describe the feasibility, usability, acceptability, and preliminary implementation impact of the combined registry training system as a prevention and monitoring model.

Performance indicators will include average reporting time, percentage of fully completed registry forms, proportion of reports with documented follow-up, proportion of cases with activation of child protection pathways, number of duplicate or repeat reports, and rate of active participation of professionals in the platform.

The elaboration of these data will provide a solid foundation for defining regional quality benchmarks in the management of suspected child maltreatment cases, identifying replicable best practices and areas for improvement. Quantitative and qualitative results, interpreted in an integrated manner, will also validate the project’s methodological model, confirming its feasibility and transferability to other regional or national contexts.

From a scientific standpoint, Sentinel will produce preliminary evidence on the feasibility of digital registries in pediatrics and on the contribution of multidisciplinary training to early detection and standardized documentation of suspected child maltreatment. The data collected will be disseminated through reports and peer-reviewed publications, contributing to the international literature on child protection and digital health.

### System-Level Impact and Future Perspectives

Beyond its immediate outcomes, the Sentinel project aims to produce a sustainable and long-term impact on the regional health and social care systems.

From an organizational perspective, the adoption of the digital registry and the establishment of a network of trained professionals are expected to strengthen structural collaboration between hospitals and community services, promoting information interoperability, case traceability, and timeliness of interventions.

In the medium term, the project is expected to reduce the latency between suspicion and formal reporting of abuse, improving the promptness of protective interventions and potentially reducing the cumulative harm experienced by affected children. Standardized data collection will facilitate communication with social services and the juvenile judiciary, ensuring more complete, coherent, and evidence-based reporting.

In the long term, the model could be scaled up and adapted to other regions, paving the way for the establishment of a permanent regional child maltreatment registry and, subsequently, a national surveillance network. This process will provide updated and comparable epidemiological data essential for policy planning, resource allocation, and evaluation of child protection strategies.

Finally, the integrated approach of Sentinel is expected to foster a broad cultural shift toward child protection, promoting a shared culture of interdisciplinary collaboration, scientific evidence, and collective responsibility. The project thus transcends its technical outcomes, aiming to achieve a systemic transformation in how the health care system identifies and responds to child vulnerability—restoring pediatrics’ central role in primary prevention and public health promotion.

## Discussion

### Principal Findings

The Sentinel project represents an innovative pilot model for strengthening the health care response to suspected child maltreatment. Its main strength lies in the synergy among digital technology, professional training, and collaboration between hospital and community services. This integration addresses a long-standing gap in the health care system: the difficulty of coordinating information, standardizing reporting procedures, and ensuring timely communication among agencies involved in child protection. By combining these components within a single operational framework, the project aims to improve both the recognition and management of suspected maltreatment cases.

The main study output will be the evaluation of a REDCap-based registry [45], conceived not merely as a data collection tool but also as a clinical and governance instrument. Through a secure, standardized, and auditable system, the registry will integrate nonidentifiable clinical, psychological, social, and pathway-related data on suspected maltreatment. Such standardization is intended to reduce information fragmentation, improve the timeliness and accuracy of documentation, and enable the generation of epidemiological and quality indicators for regional and national planning. Beyond its operational purpose, the registry will serve as a research infrastructure, providing real-world evidence on suspected or confirmed cases of maltreatment. It will offer a solid basis for studies on risk factors, diagnostic pathways, and outcomes of multidisciplinary interventions. The registry structure will also enable longitudinal analyses, allowing for the identification of temporal trends and the development of predictive models of risk within vulnerable populations.

In parallel, the training component represents the transformative core of Sentinel. Through an integrated theoretical-practical approach, the training program aims to strengthen the clinical, communicative, and relational competencies necessary to recognize early signs of abuse and manage complex cases using a trauma-informed and coordinated framework. The program promotes the translation of knowledge into practice, combining didactic sessions with multidisciplinary discussion and simulation-based learning. Participants are expected to gain a deeper understanding of child protection procedures; legal obligations; and the mechanisms of cooperation among health, social, and judicial systems.

A key element of the project is its ability to bridge applied research and clinical practice improvement, creating an operational ecosystem in which clinical, social, and educational data converge to support evidence-based decision-making [47,48]. The integration of registry data with training outcomes will allow for both quantitative and qualitative evaluation of how professional competence enhancement directly influences reporting behaviors and child protection outcomes, an area still underexplored in the literature on child maltreatment prevention. This combined approach strengthens the translational value of the project by linking professional education, digital documentation, and measurable changes in clinical practice.

The most distinctive added value of Sentinel lies in the establishment of a network of “Sentinel pediatricians,” trained and empowered professionals capable of early identification and surveillance of suspected abuse cases [47]. This network will serve as a sustainable, self-replicating resource within the health care system, ensuring the continuity of protective activities beyond the project’s duration [49,50]. Through systematic use of the digital registry and shared protocols, Sentinel pediatricians are expected to reduce the latency between suspicion and formal reporting, improving both the timeliness and overall effectiveness of the protection system [51]. The Sentinel project thus combines technological innovation, educational empowerment, and organizational coordination to produce measurable and lasting improvements in the recognition and management of child maltreatment. Validation of this integrated model of surveillance and professional development will provide a replicable framework for future regional and national implementations, marking a concrete step toward a more effective, evidence-based, and culturally aware child protection system [52].

### Comparison With Prior Work

International literature supports the effectiveness of integrating digital systems with structured educational programs to improve detection of child abuse cases and promote evidence-based clinical practices. Previous studies have demonstrated that targeted training programs significantly increase health care professionals’ ability to recognize and report suspected maltreatment [37-39]. Similarly, the implementation of digital registries in pediatric and social care contexts has been shown to enhance case traceability, data quality, and interprofessional collaboration [53,54]. The Sentinel project builds on this evidence but adds a distinctive integrative dimension: the synergistic combination of training and technology within a single, empirically tested surveillance platform. This hybrid structure, combined with the use of validated evaluation tools such as the SUS and advanced statistical modeling, positions Sentinel as a replicable and scalable best practice model adaptable to different regional or national contexts. Moreover, compared with prior initiatives, Sentinel introduces a more methodologically rigorous approach to assessing training effectiveness, integrating quantitative (pretest-posttest score changes and effect sizes) and qualitative (thematic analysis of participant feedback) metrics to evaluate the true impact of the intervention.

### Limitations

Despite its innovative potential, the project has several limitations that warrant careful consideration. First, its monocentric and pilot nature limits the generalizability of the findings, which will need confirmation in larger and more diverse settings. Data collection, carried out by a relatively small group of professionals, may be influenced by organizational or individual variables such as workload and familiarity with digital tools.

Another limitation concerns the quality and completeness of registry data. As the registry is designed as a very simple tool, the reliability of the information will depend even more on participants’ consistency and engagement, reinforcing the need for basic but continuous supervision and periodic checks. Similarly, the effectiveness of training may vary according to participants’ motivation and contextual differences between hospital and community settings. Finally, the relatively short project duration precludes comprehensive evaluation of long-term effects, particularly regarding the sustainability of the network and the retention of acquired skills. To consolidate the results, follow-up assessments and ongoing professional development programs will be required.

### Conclusions

The Sentinel project represents a strategic step toward a more structured approach to the prevention, monitoring, and management of suspected child maltreatment. By combining standardized data collection with continuous professional development, it supports the creation of an informed, coordinated, and responsive pediatric network. Its methodological rigor, ethical safeguards, and scalability make it a valuable pilot initiative for public health. Future developments may include expansion to additional regional contexts and the establishment of a regional or national observatory aimed at consolidating data, sharing best practices, and informing evidence-based policies. Ultimately, Sentinel goes beyond a technical or educational intervention. It proposes a cultural shift in which child protection is understood as a shared responsibility of pediatric and public health medicine grounded in knowledge, accountability, and interdisciplinary collaboration.

## Abbreviations

CET: Comitato Etico Territoriale
SPIRIT: Standard Protocol Items: Recommendations for Interventional Trials
SUS: System Usability Scale
